# Re: Non-progressive breast carcinomas detected at mammography screening: a population study—a model test or a novel test of cancer regression?

**DOI:** 10.1186/s13058-023-01708-2

**Published:** 2023-09-20

**Authors:** Per-Henrik Zahl

**Affiliations:** Norwegian Institute of Public Health, Oslo, Norway

Dear Editor, 

Heggland and colleagues [1] have presented a very interesting Norwegian data set and analysed the data using an age-period-cohort model. The authors conclude that nearly one in six breast carcinomas detected at screening may be non-progressive.

Age-period-cohort models are overparametrized and therefore very speculative by nature and need to be validated by model checking [2]. At age 69 years, the excess incidence rate is 1614 per 100,000 women in the model. If the women in this cohort had not attended screening for 20 years, these tumours should accumulate in the absence of screening and tumours should be detectable at a prevalence screening at age 69 years.

There are numerous studies on the detection rate of previously unscreened women we can use to validate the model by Heggland and colleagues [1]. When public biennial mammography screening started in Norway in 1996, there were 129 invasive breast tumours among 13,749 women aged 68–69 years in 1996–97, whereof 82 tumours were detected at mammography screening [3]. Assuming 80% attendance rate, this yields an age-specific detection rate of (100,000 × 82/13,749)/0.80 = 746 invasive breast cancers among 100,000 screened women. In addition, there were about 160 DCIS detected. That yields a detection rate of about 900 per 100,000 screened women.

When comparing with the cumulative excess incidence rate at age 69 years in the model, we get that about 900 of 1614 tumours (55%) are actually detectable, and 45% of the tumours have disappeared. This is actually a novel test of cancer regression. Note that cancer regression has indirectly been reported in a randomized mammography screening trial too [4]—about 50% of MRI detected tumours most likely regressed in this study.

Low sensitivity is an alternative explanation to cancer regression we have to consider. The incidence increase from age 50 to 69 years is almost linear. That means that on average tumours have been detectable by mammography for 10 years at age 69 years in this model. Assuming 45% of the tumours do not grow before age 69 and are not detected during multiple screening sessions, and then start growing after age 69 years and become clinical is unjustified.

There are some very strong biological evidence that small tumours may go in spontaneous regression. Some cancers exist in an equilibrium with the immune system. The Bacillus Calmette-Guérin (BCG) vaccine for tuberculosis has been used to treat high-risk urinary bladder tumours as well as malignant melanoma for over 50 years [5]. Indeed, modern immune therapy is based on interaction with the immune system. More recently, pembrolizumab has been approved by FDA for treatment of high-risk non-advanced and advanced-stage triple-negative breast cancer [6].

It is important to consider cancer regression when discussing overdiagnosis when the overdiagnosis model do not fit data.

## Data Availability

Aggregated data being used are published in the references or can be accessed from The Cancer Registry of Norway.
